# Spatiotemporal trends in severe complicated influenza among the local population in Taiwan region, 2003-2023

**DOI:** 10.4178/epih.e2025016

**Published:** 2025-04-02

**Authors:** Kangjun Wu, Yujian Lu

**Affiliations:** 1 Comprehensive Management Office, Zhenhai District Center for Disease Control and Prevention, Ningbo, China; 2 Department of Public Health Laboratory Sciences, Zhenhai District Center for Disease Control and Prevention, Ningbo, China

**Keywords:** Incidence, Severe influenza, Epidemiology

## Abstract

**OBJECTIVES:**

Severe influenza has raised considerable concern worldwide, and its incidence appears to have shifted in the context of globalization. This study aimed to examine the temporal, spatial, and demographic distributions of local severe influenza cases in Taiwan region from January 2003 to June 2023.

**METHODS:**

We aggregated severe complicated influenza cases by month, area (city/county), age, and sex. The age-standardized incidence rate (ASIR) was calculated to compare differences across regions and populations. Yearly incidence rate ratios comparing males to females were also computed to assess sex differences.

**RESULTS:**

A total of 16,459 cases were included from 2003 to 2023. Crude incidence rates per 100,000 population were 0.07-0.14 for 2003-2008, 3.64-9.81 for 2009-2019, and 0.00-1.87 for 2020-2023. Higher incidence rates were observed in Hualien and Taitung Counties, with average ASIRs exceeding 10.00 per 100,000 population, compared to other cities. Except for 2005 and 2007, the incidence among males exceeded that among females, with ASIR ratios ranging from 1.10 to 2.20. The highest incidence was observed among populations aged 0-4 and those aged ≥55.

**CONCLUSIONS:**

The incidence of severe complicated influenza exhibited clear regional and demographic variations in Taiwan region. The observed rebound in incidence calls for increased vigilance to protect vulnerable populations from severe illness.

## INTRODUCTION

The influenza virus, a single-stranded negative-sense segmented RNA virus, exhibits high genomic variability, which facilitates its transmission among both avian and human populations. Dominant influenza virus strains have also undergone significant changes over time. Since the beginning of the 21st century, multiple influenza pandemics and epidemics have occurred [1]. Globally, influenza is estimated to cause 1 billion cases annually, with 3-5 million of these resulting in severe illness [2]. Case fatality rates among severely ill patients range from approximately 1% to 22% [3]. The global incidence of severe influenza is alarmingly high; a meta-analysis reported an influenza-related hospitalization rate of 40.5 per 100,000 population [4]. In Taiwan region, mild influenza cases are not required to be reported individually; however, influenza cases with severe complications—referred to as severe complicated influenza—are reported as class IV notifiable infectious diseases by health professionals [5]. From 2011 to 2020, influenza-related fatalities accounted for 40% of severe complicated influenza cases in Taiwan region [5]. During the 2022-2023 season, however, the influenza vaccination rate was only 32.5% among individuals aged ≥50 years, and approximately 50% among those aged ≥65 years [6]. The coronavirus disease 2019 (COVID-19) pandemic disrupted influenza transmission both in Taiwan region and worldwide [7-9], yet a rebound in influenza activity—marked by unusual patterns and hemispheric disparities—was observed from the first half of 2022 [10]. FluNet statistics indicate that the global influenza positivity rate peaked at 24.1% in week 51 of 2022, followed by another prolonged epidemic peak at the end of 2023 [11]. Aligning with global trends, surveillance data also indicate a notable surge in influenza positive detection rates in Taiwan region during early 2023. Before 2023, the predominant virus was the influenza A (H3 subtype), which later transitioned to a co-circulation of influenza A (H3 and H1 subtypes) [12]. Surveillance of severe complicated influenza is instrumental in characterizing influenza-related hospitalizations and informing healthcare strategies and medical decision-making.

In this study, we conducted a cross-sectional analysis of local severe complicated influenza cases in Taiwan region. Our primary objective was to characterize the temporal, spatial, and demographic distributions of severe complicated influenza in Taiwan region from 2003 to the first half of 2023.

## MATERIALS AND METHODS

### Data sources

Monthly aggregated data for confirmed severe complicated influenza cases in Taiwan region from January 2003 to June 2023 were obtained from the Taiwan Center for Diseases Control Open Data Portal (https://data.cdc.gov.tw/en/). The data were aggregated by sex, age, and area (county/town) for each month, and sourced from the Notifiable Infectious Disease Report System (NIDRS). In Taiwan region, when a patient seeks medical attention and their symptoms or laboratory test results meet the criteria for infectious disease reporting—or when a physician deems it necessary—the case is reported to the NIDRS either directly by the physician or via the hospital’s infection control team or a designated individual. The reporting definition for severe complicated influenza includes individuals who require intensive care unit (ICU) treatment (or hospitalization before August 2014) or who die within 2 weeks of developing influenza-like illness (ILI) due to complications such as pulmonary and neurological manifestations, invasive bacterial infections, myocarditis, or pericarditis. Furthermore, ILI cases must meet the following 3 criteria: (1) sudden onset of fever (ear temperature ≥38°C) accompanied by respiratory symptoms; (2) the presence of muscle pain, headache, or extreme fatigue; and (3) the exclusion of milder conditions such as a runny nose, tonsillitis, or bronchitis. Cases meeting these criteria must be submitted through the NIDRS or via a written report within 1-week. After a case is reported, the local health authority regularly tracks and updates its ICU admission and mortality status, and subsequent laboratory test results are used to confirm the case. Confirmed cases of severe complicated influenza must meet the reporting definition and satisfy one of the following criteria: (1) isolation and identification of the influenza virus from respiratory clinical specimens (e.g., throat swabs); (2) a positive molecular nucleic acid test result from clinical specimens; (3) a positive antigen test result from clinical specimens; or (4) a positive serological antibody test result, showing a ≥4-fold increase in antibody titer between the acute and convalescent phases [13]. Only confirmed cases were accessible in the open database.

Annual population data from 2003 to 2022 (and monthly population data for January to June 2023) were obtained from the Taiwan Department of Household Registration (https://www.ris.gov.tw/). This data was disaggregated by sex, age, and area (county/city) for each year.

### Statistical analysis

Local confirmed severe complicated influenza case data were tabulated by month, city/county, sex, and age group (using 5-year age categories from 0 to 69 years and a final category for ≥70 years, totaling 15 groups). The crude incidence rate (per 100,000 population) was calculated to display the actual local incidence. To compare incidence variations among regions and periods, we computed the age-standardized incidence rate (ASIR; per 100,000 population) by summing the product of each age group’s crude incidence rate and its corresponding weight (ωᵢ), derived from the new World Health Organization (2000-2025) world standard population (https://seer.cancer.gov/stdpopulations/world.who.html). The relevant equations are as follows:


(1) $$ASIR=\sum_{i=1}^{15}Crude\;incidence\;rate_{i}\times \omega_{i}$$

(2) $$Crude\;incidence\;rate_{i}=\frac{Count_{i}}{Population_{i}}\times 100,000$$

(3) $$\omega_{i}=\frac{Standard\;population_{i}}{\sum_{i=1}^{15}Standard\;population_{i}}$$

The geographic distribution of incidence was visualized using thematic maps. Sex differences in incidence were quantified by calculating the ratio of the male to the female incidence rates. A polynomial function was employed to assess the trend of incidence across age groups—treated as a numerical variable—using a generalized additive model with a quasi-Poisson distribution link function. The basic model was specified as follows:


ln(Cases)~ln(Population)+ploy(Age groups, degree)+s(Year)+factor(Sex)

In this model, temporal trends and sex differences were controlled using spline functions and factor variables, respectively. The degree of the “poly” function corresponded to the highest power of the variable in the polynomial expression.

The base map for visualizing incidence data was compiled from the Taiwan Infectious Disease Statistics System (https://nidss.cdc.gov.tw/en/nndss/DiseaseMap?id=487a). All data mining, processing, and visualization were performed using Microsoft Excel version 2309 (Microsoft 365, Redmond, WA, USA), and R version 4.3.1 (R Foundation for Statistical Computing, Vienna, Austria). Thematic maps were generated and merged as scalable vector graphics (SVG) documents using R version 4.3.1, SVG code, and Inkscape version 1.3 (https://inkscape.org/).

### Ethics statement

Not applicable due to only using public aggregated data in this study.

## RESULTS

### Overview of severe complicated influenza cases

**Figure 1.**
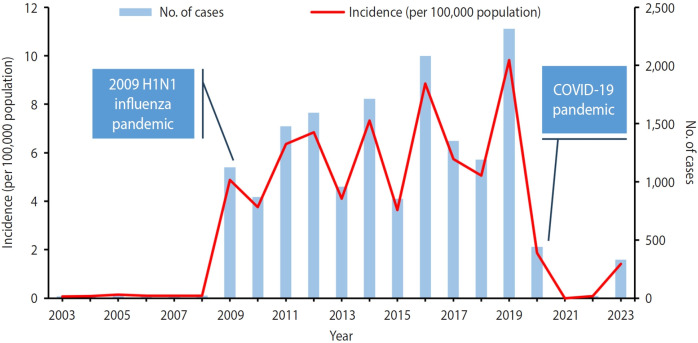
Annual number and incidence of severe complicated influenza cases in 2003-2023. For 2023, only cases with onset from January to June are included. Bars represent the number of cases, while the line depicts the crude incidence rate. The study period is divided into 3 segments: pre-2009 H1N1 pandemic (2003-2008), during and post-2009 H1N1 pandemic (2009-2019), and the coronavirus disease 2019 (COVID-19) pandemic (2020-2023).

From January 2003 to June 2023, there were 16,459 locally confirmed cases of severe complicated influenza. Overall, the male-to-female ratio was 1.34:1.00. Cases among individuals aged ≥55 comprised more than 60% of the total, while other age groups accounted for only 1.7-6.6% (Table 1). Detailed yearly characteristics are provided in the Supplementary Material 1. The annual crude incidence rate ranged from 0.00 to 9.81 per 100,000 population (mean rate, 3.35), and the incidence trends can be categorized into 3 distinct periods (Figure 1). In 2003-2008 and 2021-2022, the annual incidence rate was considerably lower (0.00-0.14 per 100,000 population) than during 2009-2019 (3.64-9.81 per 100,000 population). Additionally, the annual incidence rates were 1.87 per 100,000 in 2020 and 1.42 per 100,000 from January 2023 to June 2023.

**Figure 2.**
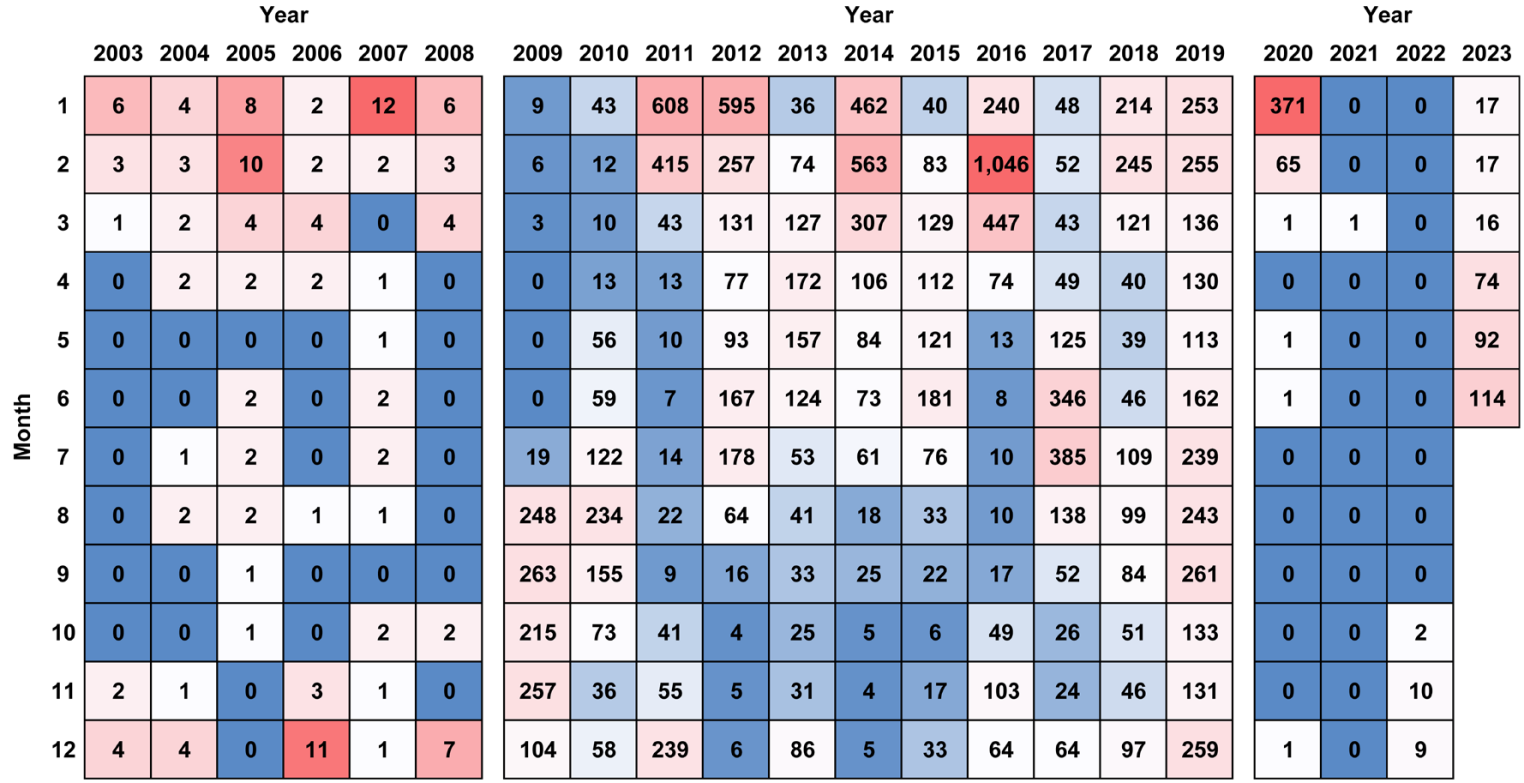
Matrix of monthly severe complicated influenza cases (2003-2023). Each cell shows monthly case counts. Red and blue grids indicate hotspots and cold spots, respectively; the deeper the red (or blue), the higher (or lower) the number of cases. Due to disparities in case counts throughout the study period, the matrix is divided into 3 separate periods. The hotspot and cold spot designations apply only within their corresponding period.

The monthly distribution of severe complicated influenza cases revealed that onset peaks predominantly occurred during winter (December, January, and February). However, additional peaks were observed in 2009-2010, 2013, 2017, and 2019. Case numbers declined between 2020 and 2023, until a notable increase was observed in April 2023 (Figure 2).

### Incidence by county/city, sex, and age

**Figure 3.**
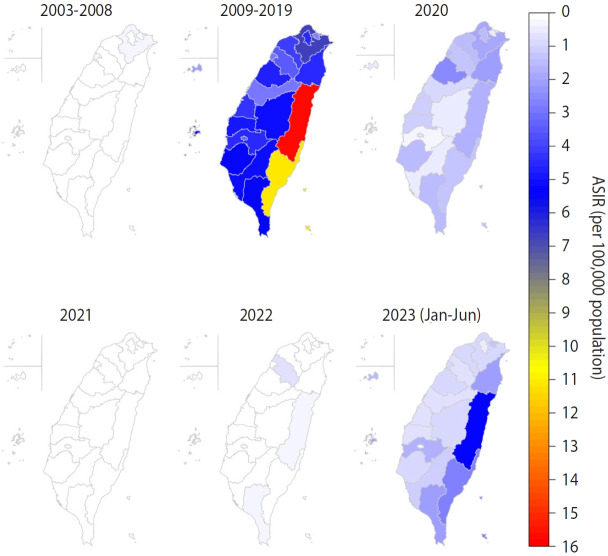
Spatial distribution of severe complicated influenza by period. For 2003-2008 and 2009-2019, the average annual age-standardized incidence rates (ASIR) are displayed. For 2020-2022 and 2023, both the annual ASIR and the cumulative incidence rate (from January to June) are shown.

In addition to the temporal variations, substantial regional disparities in the incidence of severe complicated influenza were observed (Figure 3). High ASIRs were predominantly seen in the eastern regions, with average annual ASIRs of 15.70 and 11.11 per 100,000 population in Hualien County and Taitung County, respectively, during 2009-2019. From January 2023 to June 2023, the ASIR in Hualien County continued to rise, reaching levels similar to those of previous years.

**Figure 4.**
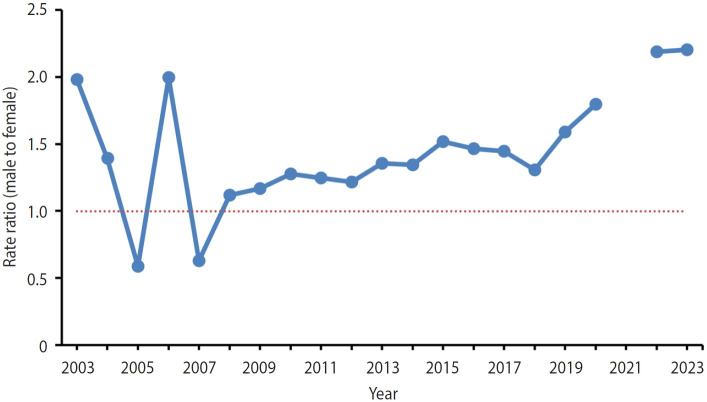
Age-standardized incidence rate ratio (male to female) of severe complicated influenza in 2003-2023. The red dashed line indicates a ratio of 1.00. No value is shown for 2021 because the numerator was 0.

Overall, the ASIR in males exceeded that in females (Figure 4). While the male-to-female rate ratios were 0.59 and 0.63 in 2005 and 2007, respectively, they ranged from 1.12 to 2.20 in other years, showing an upward trend. In 2021, the rate ratio was not calculated due to a zero ASIR in females.

**Figure 5.**
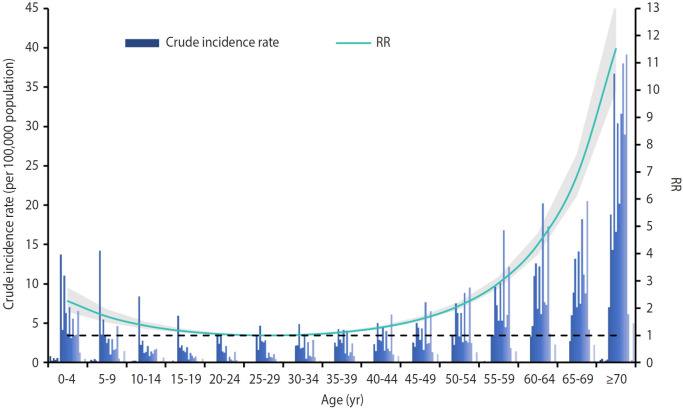
Crude incidence rate of severe complicated influenza and its association with age groups. Each set of bars represents the crude incidence rates for a specific age group, with individual bars showing the rates for each year from 2003 to 2023. A generalized additive model was used to examine the relationship between age groups (using the 25-29-year age group as the reference) and crude incidence rates, adjusting for year. The curve and shaded area represent the relative risk (RR) values and their 95% confidence intervals as they change with age.

Clearly, incidence rates varied not only annually but also significantly across different age groups. The crude incidence rate initially decreased with age and then increased, resulting in a J-shaped association between crude incidence and age (Figure 5). Specifically, compared to the reference group of individuals aged 25-29, those aged ≥70 exhibited a substantially higher risk (relative risk, 11.52; 95% confidence interval, 9.98 to 13.30).

## DISCUSSION

In this study, we conducted a comprehensive temporal, spatial, and demographic analysis of severe complicated influenza incidence among the local population in Taiwan region. Our findings indicate that, based on surveillance data, the incidence of severe complicated influenza was generally higher in males compared to females during both peak and off-peak periods. However, other studies have reported differing results. For example, data from the Global Influenza Hospital Surveillance Network identified female sex as a risk factor for influenza-associated deaths and ICU admissions [14], whereas other studies have reported contrasting findings [15]. These discrepancies may be partly due to differences in the age distributions of study populations. Moreover, sex steroids likely play an important role in determining influenza severity across age and sex [16]. A human serology study found that adult females aged 18-45 exhibited greater vaccine-induced antibody responses than their male counterparts, potentially reducing the risk of future infections [17]. Regarding the molecular mechanisms underlying sex differences, both endogenous and exogenous estrogens and progestins may modulate proinflammatory responses and pulmonary inflammation, thereby enhancing antibody responses or reducing tissue damage [18,19].

The incidence of severe complicated influenza peaked among adults aged ≥55, particularly those aged ≥70, with a secondary peak observed in the 0-4-year age group. Although variations in age distribution exist, similar trends have been documented in previous studies [20,21]. In terms of immune function, both older adults and infants exhibit compromised immunity—aging in the former and immaturity in the latter—making it difficult for them to effectively combat influenza viruses [22]. While influenza vaccination can attenuate illness severity [23], vaccination coverage varies by age group. Only about one-third of individuals aged ≥50 in Taiwan region received the vaccine, with vaccination rates falling below 20% among those aged 50-64 [6]. In contrast, children aged 6 months to 3 years had a higher vaccination rate of 68.1%, particularly among those previously vaccinated [6]. Due to their propensity for genetic recombination, influenza viruses frequently alter their pathogenicity and susceptibility in humans, including shifts in circulating strains and instances of zoonotic transmission. Consequently, temporal changes in incidence also reflect viral mutations.

Severe complicated influenza typically peaked during the winter months, although abnormal peaks were noted in other months. Vaccination strategies may influence the timing of influenza peaks [24]. A comparative study in Europe demonstrated that higher influenza vaccination coverage reduced the likelihood of severe influenza-related outcomes and ICU admissions [25]. While influenza seasons generally extend from October to April in the Northern Hemisphere and from April to October in the Southern Hemisphere [26], seasonality becomes more variable with increasing distance from the equator [27]. In Taiwan’s subtropical climate, influenced by tropical conditions, influenza exhibits seasonal patterns that do not align neatly with temperature cycles. Overall, influenza transmission is strongly affected by atmospheric conditions [28], with both high and low ambient temperatures exacerbating outbreaks [29]. Additionally, the combined effects of temperature and humidity contribute to regional influenza activity patterns, as supported by epidemiological and *in vivo* studies [30].

Our study identified 3 distinct periods: 2003-2008, 2009-2019, and 2020-2023. Prior to the 2009 pandemic caused by the influenza A (H1N1 pdm09) virus, incidence rates remained low—likely due to the predominance of less virulent strains. However, the period from 2009 to 2019 saw a surge in cases. For example, in Spain the influenza A (H1N1 pdm09) virus was associated with a higher risk of severe outcomes (ICU admission and/or death) compared with influenza A (H3N2) or B viruses [31]. Nevertheless, the severity associated with different influenza virus types has varied across studies and regions [32]. The sensitivity of the surveillance system depends on the case definition employed [33], particularly regarding temporal variations in inclusion criteria: cases were defined as those requiring hospitalization prior to August 2014, whereas the threshold was elevated to necessitate ICU admission thereafter [5]. Extensive non-pharmaceutical interventions during the COVID-19 pandemic led to a global reduction in respiratory infections, including influenza, respiratory syncytial virus, and adenovirus [34]. Moreover, the pandemic impacted surveillance systems in terms of reporting completeness, sensitivity, and population representativeness [35,36]. Consistent with global trends, the incidence of severe complicated influenza was notably lower in 2020-2021 but increased steadily in 2022-2023. Spatial differences in incidence were also evident; factors such as population composition, human development index, and natural environment contribute to regional variability [37,38]. Additionally, racial disparities have led to significant differences at spatial or administrative levels [39]. Similar to age and sex differences, geographic variations are also influenced by regional vaccination coverage. Further in-depth evaluations are urgently needed to address spatial disparities and elevated incidence in specific regions.

In conclusion, the incidence of severe complicated influenza was high among males aged ≤4 and ≥55 in eastern Taiwan during 2009-2019. A significant rebound in incidence was observed in 2022-2023 following the normalization of the COVID-19 epidemic. It remains imperative to maintain vigilance against future influenza epidemics and to implement targeted health policy support for high-risk areas and populations. Increasing vaccination rates among the elderly and children continues to be the most effective strategy to reduce the disease burden associated with severe cases, particularly in regions currently experiencing higher incidence rates.

## Figures and Tables

**Table 1.** The characteristics of severe complicated influenza cases in 2003-2023^1^

Characteristics	n (%)
Total	16,459 (100)
Sex	
Male	9,413 (57.2)
Female	7,046 (42.8)
Age (yr)	
0-4	730 (4.4)
5-9	543 (3.3)
10-14	363 (2.2)
15-19	304 (1.8)
20-24	280 (1.7)
25-29	403 (2.4)
30-34	479 (2.9)
35-39	564 (3.4)
40-44	669 (4.1)
45-49	827 (5.0)
50-54	1,086 (6.6)
55-59	1,494 (9.1)
60-64	1,639 (10.0)
65-69	1,272 (7.7)
≥70	5,806 (35.3)

1 Due to the rounding of numerical values during the calculation process, the sum of partial proportions was not equal to 100%; In 2023, only cases with onset from January to June were included.

## Supplementary materials

Supplementary Material 1. The characteristics of severe complicated influenza cases in 2003-2023 [Number (Proportion/%)]
