# Supplementary material for: Spatiotemporal trends in severe complicated influenza among the local population in Taiwan region, 2003-2023
Source: Epidemiol Health. 2025 Apr 2;47:e2025016. doi: 10.4178/epih.e2025016 (PMC12178764; doi:10.4178/epih.e2025016)
Supplement: Supplementary Material 1. — The characteristics of severe complicated influenza cases in 2003-2023 [Number (Proportion/%)] [file epih-47-e2025016-Supplementary-1.docx]

**Supplementary Material 1** The characteristics of severe complicated influenza cases in 2003-2023 [Number (Proportion/%)]

| Characteristics | Year | | | | | | |
| --- | --- | --- | --- | --- | --- | --- | --- |
|  | 2003 | 2004 | 2005 | 2006 | 2007 | 2008 | 2009 |
| Total | 16 (100) | 19 (100) | 32 (100) | 25 (100) | 25 (100) | 22 (100) | 1,124 (100) |
| Sex |  |  |  |  |  |  |  |
| Male | 11 (68.8) | 12 (63.2) | 13 (40.6) | 16 (64.0) | 11 (44.0) | 11 (50.0) | 613 (54.6) |
| Female | 5 (31.3) | 7 (36.8) | 19 (59.4) | 9 (36.0) | 14 (56.0) | 11 (50.0) | 511 (45.5) |
| Age group(years) |  |  |  |  |  |  |  |
| 0~4 | 11 (68.8) | 3 (15.8) | 7 (21.9) | 4 (16.0) | 6 (24.0) | 1 (4.5) | 139 (12.4) |
| 5~9 | 2 (12.5) | 5 (26.3) | 3 (9.4) | 6 (24.0) | 3 (12.0) | 2 (9.1) | 183 (16.3) |
| 10~14 | 1 (6.3) | 1 (5.3) | 3 (9.4) | 4 (16.0) | 4 (16.0) | 2 (9.1) | 129 (11.5) |
| 15~19 | 0 (0) | 0 (0) | 0 (0) | 4 (16.0) | 0 (0) | 1 (4.5) | 96 (8.5) |
| 20~24 | 0 (0) | 1 (5.3) | 2 (6.3) | 0 (0) | 0 (0) | 1 (4.5) | 56 (5.0) |
| 25~29 | 1 (6.3) | 0 (0) | 0 (0) | 1 (4.0) | 2 (8.0) | 2 (9.1) | 66 (5.9) |
| 30~34 | 0 (0) | 0 (0) | 2 (6.3) | 0 (0.0) | 2 (8.0) | 0 (0) | 42 (3.7) |
| 35~39 | 0 (0) | 0 (0) | 0 (0) | 1 (4.0) | 1 (4.0) | 2 (9.1) | 46 (4.1) |
| 40~44 | 0 (0) | 0 (0) | 2 (6.3) | 0 (0) | 2 (8.0) | 0 (0) | 44 (3.9) |
| 45~49 | 1 (6.3) | 1 (5.3) | 1 (3.1) | 2 (8.0) | 0 (0) | 3 (13.6) | 48 (4.3) |
| 50~54 | 0 (0) | 2 (10.5) | 2 (6.3) | 1 (4.0) | 0 (0) | 1 (4.5) | 58 (5.2) |
| 55~59 | 0 (0) | 0 (0) | 2 (6.3) | 0 (0) | 0 (0) | 1 (4.5) | 50 (4.4) |
| 60~64 | 0 (0) | 1 (5.3) | 1 (3.1) | 0 (0) | 0 (0) | 0 (0) | 29 (2.6) |
| 65~69 | 0 (0) | 0 (0) | 0 (0) | 1 (4.0) | 1 (4.0) | 0 (0) | 21 (1.9) |
| ≥ 70 | 0 (0) | 5 (26.3) | 7 (21.9) | 1 (4.0) | 4 (16.0) | 6 (27.3) | 117 (10.4) |

Note: Due to the rounding of numerical values during the calculation process, the sum of partial proportions was not equal to 100%.

**Supplementary Material 1** (continued) The characteristics of severe complicated influenza cases in 2003-2023 [Number (Proportion/%)]

| Characteristics | Year | | | | | | |
| --- | --- | --- | --- | --- | --- | --- | --- |
|  | 2010 | 2011 | 2012 | 2013 | 2014 | 2015 | 2016 |
| Total | 871 (100) | 1,476 (100) | 1,593 (100) | 959 (100) | 1,713 (100) | 853 (100) | 2,081 (100) |
| Sex |  |  |  |  |  |  |  |
| Male | 493 (56.6) | 803 (54.4) | 862 (54.1) | 539 (56.2) | 979 (57.2) | 503 (59.0) | 1,232 (59.2) |
| Female | 378 (43.4) | 673 (45.6) | 731 (45.9) | 420 (43.8) | 734 (42.8) | 350 (41.0) | 849 (40.8) |
| Age group(years) |  |  |  |  |  |  |  |
| 0~4 | 41 (4.7) | 106 (7.2) | 61 (3.8) | 36 (3.8) | 70 (4.1) | 32 (3.8) | 59 (2.8) |
| 5~9 | 43 (4.9) | 62 (4.2) | 37 (2.3) | 26 (2.7) | 31 (1.8) | 10 (1.2) | 29 (1.4) |
| 10~14 | 34 (3.9) | 41 (2.8) | 17 (1.1) | 18 (1.9) | 27 (1.6) | 10 (1.2) | 16 (0.8) |
| 15~19 | 31 (3.6) | 35 (2.4) | 24 (1.5) | 21 (2.2) | 30 (1.8) | 6 (0.7) | 18 (0.9) |
| 20~24 | 38 (4.4) | 53 (3.6) | 23 (1.4) | 20 (2.1) | 34 (2.0) | 3 (0.4) | 12 (0.6) |
| 25~29 | 31 (3.6) | 86 (5.8) | 47 (3.0) | 43 (4.5) | 46 (2.7) | 9 (1.1) | 20 (1.0) |
| 30~34 | 44 (5.1) | 99 (6.7) | 37 (2.3) | 38 (4.0) | 69 (4.0) | 9 (1.1) | 48 (2.3) |
| 35~39 | 37 (4.2) | 77 (5.2) | 54 (3.4) | 47 (4.9) | 81 (4.7) | 23 (2.7) | 82 (3.9) |
| 40~44 | 27 (3.1) | 93 (6.3) | 53 (3.3) | 50 (5.2) | 83 (4.8) | 27 (3.2) | 72 (3.5) |
| 45~49 | 38 (4.4) | 96 (6.5) | 84 (5.3) | 54 (5.6) | 80 (4.7) | 30 (3.5) | 140 (6.7) |
| 50~54 | 40 (4.6) | 135 (9.1) | 115 (7.2) | 61 (6.4) | 118 (6.9) | 48 (5.6) | 165 (8.0) |
| 55~59 | 53 (6.1) | 153 (10.4) | 118 (7.4) | 88 (9.2) | 171 (10.0) | 92 (10.8) | 294 (14.1) |
| 60~64 | 45 (5.2) | 121 (8.2) | 155 (9.7) | 91 (9.5) | 172 (10.0) | 91 (10.7) | 311 (15.0) |
| 65~69 | 45 (5.2) | 65 (4.4) | 97 (6.1) | 54 (5.6) | 117 (6.8) | 69 (8.1) | 191 (9.2) |
| ≥ 70 | 324 (37.2) | 254 (17.2) | 671 (42.1) | 312 (32.5) | 584 (34.1) | 394 (46.2) | 624 (30.0) |

**Supplementary Material 1** (continued) The characteristics of severe complicated influenza cases in 2003-2023 [Number (Proportion/%)]

| Characteristics | Year | | | | | | |
| --- | --- | --- | --- | --- | --- | --- | --- |
|  | 2017 | 2018 | 2019 | 2020 | 2021 | 2022 | 2023 |
| Total | 1,352 (100) | 1,191 (100) | 2,315 (100) | 440 (100) | 1 (100) | 21 (100) | 330 (100) |
| Sex |  |  |  |  |  |  |  |
| Male | 775 (57.3) | 661 (55.5) | 1,368 (59.1) | 280 (63.6) | 1 (100) | 12 (57.1) | 218 (66.1) |
| Female | 577 (42.7) | 530 (44.5) | 947 (40.9) | 160 (36.4) | 0 (0) | 9 (42.9) | 112 (33.9) |
| Age group(years) |  |  |  |  |  |  |  |
| 0~4 | 36 (2.7) | 36 (3.0) | 65 (2.8) | 12 (2.7) | 0 (0) | 1 (4.8) | 4 (1.2) |
| 5~9 | 16 (1.2) | 17 (1.4) | 47 (2.0) | 5 (1.1) | 0 (0) | 1 (4.8) | 15 (4.5) |
| 10~14 | 12 (0.9) | 17 (1.4) | 18 (0.8) | 1 (0.2) | 0 (0) | 2 (9.5) | 6 (1.8) |
| 15~19 | 12 (0.9) | 8 (0.7) | 10 (0.4) | 2 (0.5) | 0 (0) | 1 (4.8) | 5 (1.5) |
| 20~24 | 6 (0.4) | 4 (0.3) | 21 (0.9) | 4 (0.9) | 0 (0) | 1 (4.8) | 1 (0.3) |
| 25~29 | 12 (0.9) | 10 (0.8) | 17 (0.7) | 7 (1.6) | 0 (0) | 0 (0) | 3 (0.9) |
| 30~34 | 15 (1.1) | 12 (1.0) | 47 (2.0) | 11 (2.5) | 0 (0) | 0 (0) | 4 (1.2) |
| 35~39 | 18 (1.3) | 27 (2.3) | 48 (2.1) | 16 (3.6) | 0 (0) | 2 (9.5) | 2 (0.6) |
| 40~44 | 32 (2.4) | 28 (2.4) | 119 (5.1) | 20 (4.5) | 0 (0) | 0 (0) | 17 (5.2) |
| 45~49 | 43 (3.2) | 45 (3.8) | 117 (5.1) | 23 (5.2) | 0 (0) | 1 (4.8) | 20 (6.1) |
| 50~54 | 52 (3.8) | 46 (3.9) | 173 (7.5) | 45 (10.2) | 0 (0) | 1 (4.8) | 23 (7.0) |
| 55~59 | 80 (5.9) | 109 (9.2) | 222 (9.6) | 34 (7.7) | 0 (0) | 2 (9.5) | 25 (7.6) |
| 60~64 | 120 (8.9) | 117 (9.8) | 283 (12.2) | 61 (13.9) | 0 (0) | 1 (4.8) | 40 (12.1) |
| 65~69 | 131 (9.7) | 111 (9.3) | 276 (11.9) | 59 (13.4) | 0 (0) | 0 (0) | 34 (10.3) |
| ≥ 70 | 767 (56.7) | 604 (50.7) | 852 (36.8) | 140 (31.8) | 1 (100) | 8 (38.1) | 131 (39.7) |
